# Macrophytes and their sedimentary phosphorus niche in lowland rivers

**DOI:** 10.1371/journal.pone.0330460

**Published:** 2025-09-02

**Authors:** Willem Kaijser, Verena S. Brauer, Christian Schürings, Armin W. Lorenz

**Affiliations:** 1 Faculty of Biology, Aquatic Ecology, University of Duisburg-Essen, Essen, Germany; 2 Faculty of Biology, Aquatic Microbiology, University of Duisburg-Essen, Essen, Germany; University of Limpopo - Turfloop Campus: University of Limpopo, South Africa

## Abstract

Macrophytes in lowland rivers have traditionally been studied with a focus on surface water chemistry, particularly nutrients. However, unlike in lakes, the relationship between macrophytes and surface water nutrients in rivers is generally weaker, especially in highly alkaline lowland rivers, which are often found more downstreams. In these systems, elevated sediment nutrient levels may better explain macrophyte community compositions than surface water nutrients alone. This study investigates the associations between macrophytes and sediment pore water nutrients, particularly Total Phosphorus (TP), while also considering hydromorphological factors such as flow velocity and water depth. Sampling was conducted at 76 locations in wadable lowland rivers in North Rhine-Westphalia, Germany, where macrophyte species, surface water chemistry, and sediment pore water chemistry were recorded. Relationships were analysed using Canonical Correspondence Analysis, absolute niche quantification, and a Generalised Linear Mixed Model (GLMM). Despite the potential role of pore water chemistry in macrophyte nutrient uptake, our results indicate that species niches along pore water TP did not strongly differ. Species niches extended to at least 3,000 μg L^-1^, although they preferred lower concentrations. Instead, hydromorphological variables, particularly water depth and flow velocity, exerted a stronger influence on macrophyte distribution than either surface or pore water nutrients. Tolerant species such as *Ceratophyllum demersum* and *Potamogeton crispus* were more prevalent in deeper waters with higher pH levels, while more sensitive species like *Glyceria fluitans* were found in shallower areas with lower pH levels. The GLMM estimated that the surface water TP concentrations increase by approximately 0.37% for every 1% rise in pore water TP concentrations, suggesting a notable but complex link between sediment and surface water nutrients. These findings highlight the challenges of using macrophytes as indicators of water column and pore water nutrients levels in lowland rivers. The results suggest that either these rivers are nutrient-saturated and dominated by eutrophic species, limiting their bioindication potential, or that macrophyte communities are completely impoverished. Additionally, hydromorphological alterations, such as river straightening and embankments, constrain ecotone habitats and should also be considered in successful river management strategies.

## Introduction

Macrophyte research in lowland rivers has traditionally focussed on species occurrences in relation to surface water chemistry [1–3]. Alkalinity, pH, and nutrient concentrations are commonly associated with macrophyte species distributional patterns [2–4], leading to the development of macrophyte indices for assessing the trophic state of a surface water [5–7].

However, while macrophyte-surface water nutrient relationships are well documented in lakes [8,9], they are weaker and more variables in rivers, with reported R-squared values ranging between ~0.1–0.4 [3,10,11]. This weaker relationship calls into question the reliability of macrophytes as trophic indicators in riverine environments, where multiple stressors interact [1,12,13]. The likely explanation for this difference between lakes and rivers is that, in lakes, macrophytes correlate with surface water nutrients primarily due to their influence on phytoplankton biomass, which affects light availability and thus macrophyte growth [14,15]. In rivers, this indirect relationship weakens, particularly in midstream locations, where short water residence times prevent the build-up of phytoplankton biomass and its subsequent effects on underwater light conditions [16]. While local surface water nutrients may promote epiphytic algae that can reduce light availability, grazing macroinvertebrates and allelopathic substances often mitigate this effect [17,18].

Furthermore, macrophyte-nutrient relationships appear to vary with river alkalinity. In low-alkaline rivers with siliceous substrates (e.g., sand and gravel), macrophytes exhibit relatively stronger correlations with surface water nutrients compared to high-alkaline lowland rivers, where sediments primarily consist of fine particulate organic matter and clay having high nutrient content [3]. Given that many lowland rivers are impacted by fine sediment, sediment nutrient concentrations, particularly phosphorus, may better explain macrophyte community composition than surface water alone.

Macrophytes primarily take up nutrients with their roots [19–21] making it conceivable that their distribution is more closely associated to sediment nutrient availability than to dissolved nutrients in the water column. Previous studies have found positive correlations between sediment pore water phosphorus and total macrophyte biomass [22–24]. However, beyond nutrient availability, hydromorphological factors, such as flow velocity and water depth are also known to strongly influence macrophyte distribution [25–27]. High flow velocities can physically damage and dislodge sediment-rooted macrophytes [28,29], while water depth affects light availability, favouring taller species in deeper areas. Additionally, surface water chemistry and hydrodynamics influence sediment composition in rivers with respect to grain size, which in turn selects for specific macrophyte species [25,30].

This study explored whether macrophyte species exhibit niche differentiation concerning sediment phosphorus concentrations and whether the macrophyte communities are more strongly associated with sediment pore water nutrients and hydromorphological conditions than with surface water chemistry. Specifically, we possessed the questions:

(i) Do we observe that macrophyte species exhibit distinct distributions along sediment pore water TP gradients.
(ii) Does, pore water chemistry and hydromorphological variables explain more variation in species composition than surface water chemistry, and
(iii) Is there a clear relationship between pore and surface water TP concentrations.

Exploring these questions, we aim to clarify the role of sediment nutrients in structuring macrophyte communities in high-alkaline lowland rivers and assess the implications for using macrophytes as bioindicators in these systems.

## Materials and methods

### Locations and sample procedure

**Fig 1:**
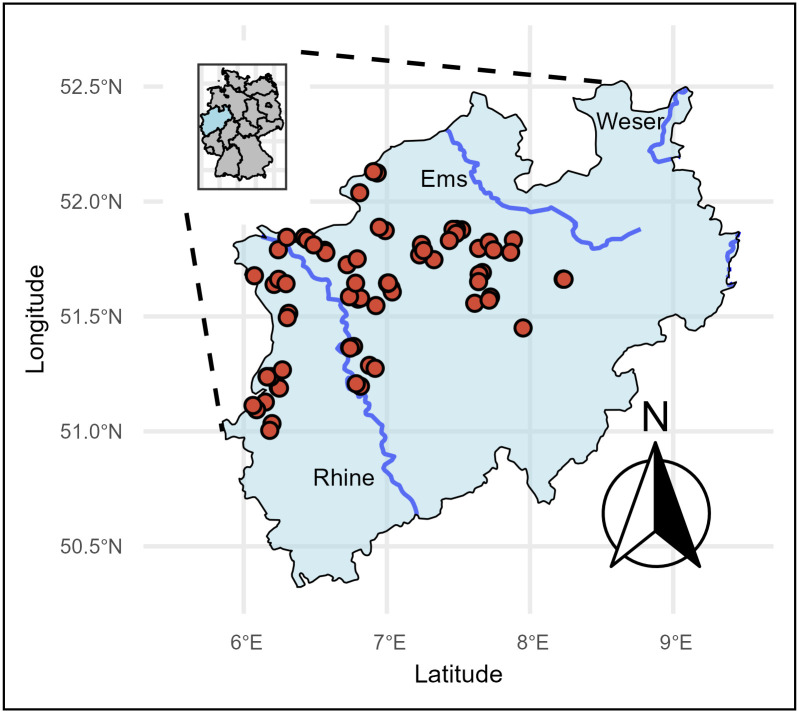
Sample locations in North Rhine-Westphalia (Germany). This figure was created by the authors in R using public domain data from the open-source rnaturalearth package [31]. It does not contain any copyrighted or proprietary map tiles or imagery. The figure is published under the CC BY 4.0 license.

Seventy-six locations within soft bottom wadable lowland rivers across North Rhine-Westphalia (Germany) were sampled between July and September of 2019 and 2020. A total of 22 taxa with 198 individual occurrences were identified. The sampling sites included streams and rivers with substrates of mud, silt, sand or small gravel (Fig 1).

At each location all macrophytes within a 100 m reach were recorded and a surface water sample was taken at the start of the reach. All taxa were identified to the species level except for *Callitriche* spp. Within each reach, a sediment sample was taken at the point where a species reached the largest density. This was done by inserting a core sampler with an inner diameter of 90 mm, to a depth of 15 cm into the sediment. These sediment samples were then placed in a 1100 ml container, from which pore water was later extracted in the laboratory. The river water and sediment samples were collected from publicly accessible, non-protected waters, without impacting red-listed species or their habitats, and therefore did not require official permission.

Hydromorphological measurements were taken at the edges of macrophyte stands. Flow velocity (Schiltknecht MC20, m s ⁻ ¹) and water depth (rounded to the nearest decimeter) were recorded at the locations with the highest values within the reach (see Table 1 for variable ranges). Additionally, Euclidean distances between each sample location were calculated based on their x and y coordinates. The row vectors for each sample location were summed, higher values indicating samples taken further away from other locations. Additionally, the distance to the river source was extracted for each sample location.

**Table 1:** Ranges, median (5%, 50% and 95%), mean and standard deviation of the sampled variables.

Metric	Velocity (m s^-1^)	Depth (m)	pH	HCO3 (mg L^-1)^	Surface water TP (ug L^-1^)	Pore water P (ug L^-1^)	Surface water NH4 (ug L^-1^)	Pore water NH4 (ug L^-1^)	Surface water NO3 (mg L^-1^)	Pore water NO3 (mg L^-1^)	Spatial distance (Euclidean)	Distance to source (km)
**5%**	0	1	7.1	62.91	11.87	35.66	0.1	0.22	0.32	0.12	120.89	1.39
**50%**	0.15	3	7.8	205.11	49.33	375.85	0.29	0.89	10.49	0.32	157	11.14
**95%**	0.53	6.15	8.2	316.5	304.37	2733.18	0.68	5.18	46.11	10.28	232.53	56.97
**Mean**	0.19	3.36	7.8	194.27	123.06	777.55	0.33	1.57	13.11	2.3	163.7	17.11
**Standard deviation**	0.18	2.05	0.4	81.54	198.49	1095.13	0.2	1.58	13.45	8.47	38.02	17.33

### Surface and pore water sample analysis

The sediment and surface water samples were transported to the laboratory for analysis. Surface water samples were filtered using a Whatman filter with a pore size of 45 μm. Pore water was extracted by inserting a Rhizon SMS 5 cm filter (pore size of 0.12–0.18 μm; Rhizosphere research products, Wageningen, Netherlands) approximately 3 cm below the sediment surface. A Rhizon is a small cylindrical filter that uses under-pressure, created by a syringe, to extract pore water from the sediment.

One portion of each water sample was acidified and stored for analysis of Total Phosphorus (TP) using Inductively Coupled Plasma Mass Spectrometry (ICP-MS, PerkinElmer). Another portion was frozen at −18 °C for later analysis of nitrate (NO_3_^-^) with ion chromatography (Metrohm) and photometric analysis (WTW PhotoLab S12) of ammonium (NH_4_^-^). Additionally, pH (measured with WTW pH 320), and bicarbonate (HCO_3_^-^) concentration of the surface water were measured on the same day of the sampling. Bicarbonate was determined by titration to a pH of 4.3, using a 716 DMS Titrino. The ranges of these variables are displayed in Table 1.

### Statistical analysis

To explore whether macrophyte species exhibit niche differentiation with respect to sediment pore water TP (Expectation i), we estimated the posterior predictive distribution of pore water TP for each species using R2jags v. 0.7–1 [32]. The posterior predictive distribution represents the probable values of a new observation given the data and prior information. Priors for each taxon and each variable were derived individually (see S1 File for rationale). The model was run for 10,000 iterations and thinned by 10 and further controlled similarly to the GLMM as will be explained below. Both the mean and standard deviation of a taxon were assumed to be generated from a Gamma distribution. The posterior predictive distribution was also assumed to follow a Gamma distribution.

To explore species variability in relation to the different environmental variables (expectation ii) a Canonical Correspondence Analysis (CCA) was applied in R using the vegan v. 2–6.4 package [33]. Before applying the CCA, the environmental data were quantile inverse rank transformed to place the positions of the species relative to each other. 

To examine the relationship between pore water and surface water TP (Expectation iii), we applied a Generalized Linear Mixed Model (GLMM) within a Bayesian framework using R2jags. Since each sample location has multiple duplicate samples of the sediment nutrients at each site, the associated variance at each site was modelled as a ‘random effect’. The GLMM had a Log-link and Gamma distributed likelihood. Pore water TP was used as the predictor variable and surface water TP as the response variable. Pore water TP was log transformed to improve visualisation, improve model fit and allow the regression coefficient to be interpreted as elasticity coefficient [34]. The prior for the intercept was a normal distribution with a mean of −1 and a standard deviation of 0.5 [N(−1, 0.5)]. The prior for the regression coefficient was set as N(0.69, 0.14). The shape parameter was set as uniformly distributed between 0 and 2 due to absence of specific information (see the supplementary material for the rationale on the prior).

The model ran for 20,000 iterations, with a burn-in of 1,000, using eight chains, thinned by 20. The chains were checked via trace plots using the mcmcplots package v. 0.8.3 [35]. Rhat was < 1.00 and effective sample size > 3,000. Regression results were presented as hypothetical outcome plots [36], which depict 500 simulated hypothetical regression lines derived from the posterior distribution. All intervals were displayed as high-density-intervals at 95%. All statistical analyses were performed in R v. 4.3.2 [37] and visualised using see v. 0.8.1 [38] and ggplot2 v. 3.3.4 [39].

## Results

**Fig 2:**
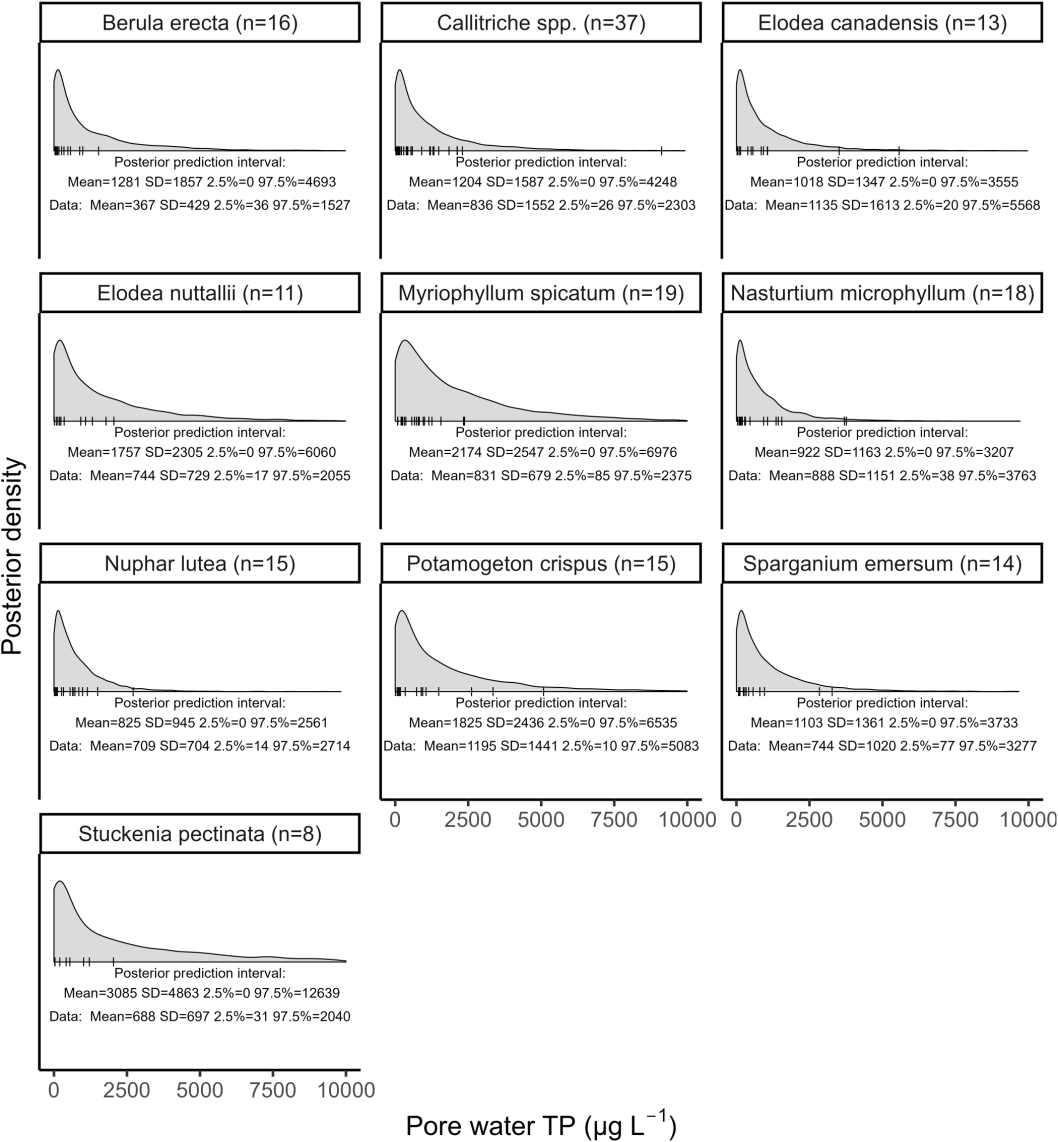
Hypothetical niches of macrophyte species for pore water TP. Grey area indicates the probability density curves of the posterior simulations. The small vertical lines indicate the observed presences of the respective species at particular pore water TP concentrations. Only species with n > 5 observations are included.

The hypothetical niche of the macrophyte species in relation to the pore water TP was simulated using the posterior prediction interval (Fig 2). While species occurrence probabilities were generally highest at lower pore water TP concentrations, the wide predictive intervals indicate that species can persist across a broad range of phosphorus levels. The predicted intervals were generally broad and largely overlapping across the pore water phosphorus gradient, indicating limited niche differentiation based on pore water TP. The widest interval was observed for *S. pectinata* with a 95% interval extending up to approximating 12,000 μg L^-1^. In contrast, the smallest interval was observed for *Nuphar lutea* with upper limit around 3,000 μg L^-1^.

**Fig 3:**
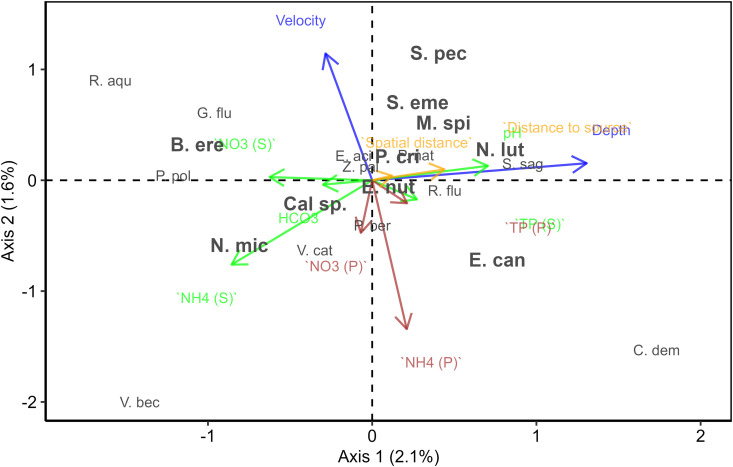
Biplot of the CCA, where the blue colour indicates hydromorphological, orange spatial, green surface water and brown pore water variables. The (S) or (P) behind a variable name indicates surface or pore water origin of the samples. Species with n > 5 observations are in bold.

The CCA applied on the dataset to distinguish the positions of species along different environmental gradients, capturing 8.6% of the variation in the community (Fig 3). Hydromorphological factors, specifically water depth, pH and flow velocity contributed more to species distribution patterns than either pore or surface nutrients. Depth was primarily associated with the x-axis, while flow velocity was aligned with the y-axis, indicating that hydromorphological variables accounted for the largest proportion of the variance in the lowland communities.

Species exhibited distinct placement in response to environmental variables. ‘Tolerant’ species clustered on the right side of the plot, while ‘sensitive’ species were positioned towards the left. Eutrophication tolerant species, such as *Ceratophyllum demersum*, *Potamogeton crispus* and *Stuckenia pectinata* were found in deeper waters, at higher pH, at higher pore and surface water TP, and further away from the source. However, both *C. demersum*, *Elodea canadensis* were positioned closer to areas with lower flow velocities. In contrast species typically found in shallower waters and upstream locations, such as *Glyceria fluitans* and *Potamogeton polygonifolius* were found at lower pH, and lower pore and surface water TP.

**Fig 4:**
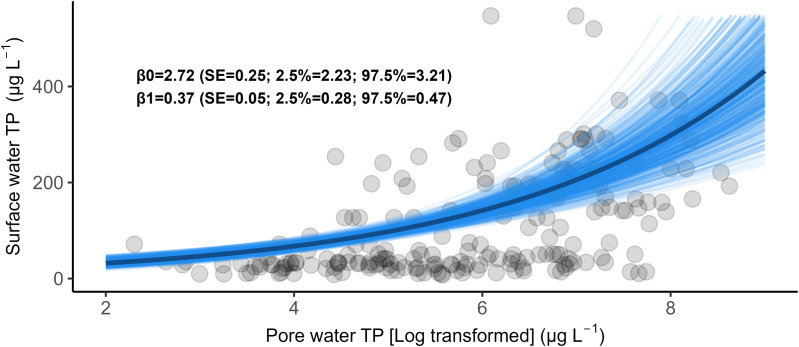
Regression of log-transformed pore water total phosphorus (TP) versus surface water TP. Grey circles show the samples, the blue line displays the expected value, black lines show the HOP lines. ß0 and ß1 show estimates of the GLMM.

A regression analysis revealed a positive relationship between pore water TP and surface water TP (Fig 4). The estimated regression coefficient (β1) indicated that a 1% increase in pore water TP corresponded to an approximate 0.37% increase in the surface water TP.

## Discussion

### Phosphorus niches

We expected a clear differentiation among macrophyte species along the pore water TP gradient. However, our results indicate that species niches were broad, spanning phosphorous concentrations between approximately 3,000–10,000 μg L^-1^. This suggests that phosphorus is not a limiting factor in these rivers, likely due to sediment saturation with nutrients. Consequently, macrophytes in these environments do not serve as reliable indicators of pore water, at least not under high phosphorus concentrations. It is possible that in rivers with lower, growth-limiting phosphorus concentrations, macrophyte distributions would exhibit a clearer relationship with pore water TP. However, in the studied lowland rivers, oversaturated nutrient conditions appear to obscure such patterns.

On the other hand, a key limitation of this study lies in the spatial mismatch between the scale of the macrophyte surveys (100 m river reach) and the use of a single sediment core taken from the densest macrophyte stand per species in each reach to assess pore water TP. Sediment composition and pore water nutrient concentrations can vary considerably within these sections, influenced by hydrodynamics and local biological activity.

However, this study was designed as an exploratory investigation broad-scale patterns between pore water TP and macrophyte community composition in riverine ecosystems. While it does not aim to assess causal relationships or test specific hypotheses, exploratory research still seeks patterns in the data that can guide future, more targeted studies. Even in exploratory contexts – such as lake monitoring, where chlorophyll-a and TP are sampled – clear patterns typically emerge if the underlying processes are strong. For chlorophyll-a and TP, such correlations are consistently observed. In contrast, our findings suggest that either (1) the nutrient-macrophyte relationship within river reaches is weak, or (2) the spatial resolution of our sampling design was insufficient to detect existing patterns.

These hypotheses require further investigation, for instance through mesocosm or germination experiments. One possible explanation for the lack of niche differentiation is that macrophyte assemblages in these lowland rivers are already impoverished, with only eutraphentic species persisting. The dominance of ‘tolerant’ species [5,6,40] suggests that more sensitive taxa may have disappeared early in the degradation process and have been unable to recolonize. Re-establishment of diverse macrophyte communities may be hindered by persistent eutrophic conditions, competitive exclusion, and the loss of suitable ecotone habitats further downstream.

The later could be assessed in the future studies using germination experiments to determine which species still remain in the seed banks, as well as transplant experiments to test whether sensitive species can survive at sites where they are currently absent. Additionally, more spatially detailed and controlled sediment sampling – such as overlaying a grid-based design across each river reach – could improve our ability to resolve within-reach variation. Such methodological refinements, in combination with experimental approaches, will be essential for deepening our understanding of nutrient – vegetation interactions in river ecosystems.

### Influence of hydromorphological variables

We expected that macrophyte species distribution would be strongly related to both pore water chemistry and hydromorphology, with surface water chemistry playing a minor role. However, our results indicate that water depth and flow were the most influential factors, captured by the first two axes of the CCA.

Macrophyte species found in deeper waters and at higher pH levels such as *M. spicatum*, *P. crispus*, *Sparganium emersum* and *S. pectinata* are typically located further downstream in larger and deeper rivers [41]. These species are capable of forming canopies, allowing them to outcompete small species like *G. fluitans* or *Callitriche* spp., which are associated with shallow upstream rivers [15,41].

Additionally, along the upstream-downstream gradient, primary producers take up CO_2_, leading to an increased pH, likely a proxy for CO_2_ limitation [4,42]. Flow velocity also strongly influenced macrophyte distribution. Species characterised by streamlined growth forms and strong root systems, such as *G. fluitans*, *M. spicatum*, *S. emersum* and *S. pectinata*, are positioned at the top half of the plot [29,43]. These findings underscore the importance of hydromorphological factors in structuring macrophyte communities, highlighting that river management and restoration efforts must consider not only nutrient conditions but also hydromorphological alterations, such as flow velocity and depth structuring ecotone areas.

### Relationship between pore water and surface water total phosphorus

Our study aimed at investigating the relationship between pore water and surface water Total Phosphorus (TP) concentrations in lowland rivers, a topic for which quantitative information remains limited in literature. We found that surface water TP concentrations increased by approximately 0.37% for every 1% increase in pore water TP concentrations. This relationship is strong, especially when compared to the approximately 1.0 (1%) relationship between log(chlorophyll-a) and TP reported by Phillips et al. [14] and the −0.32 (0.32%) decline in macrophytes species richness relative to an increase in chlorophyll-a concentrations [44]. In contrast, Schneider and Melzer [45] found no clear correlation, possibly due to differences in methodology and analysis.

If the relationship between pore water and surface water TP is a fundamental characteristic of rivers, future studies investigating macrophyte responses to surface water nutrients must account for this relationship, as detected correlations may not necessarily indicate direct casual links. During summer periods with low to moderate discharge – conditions increasingly intensified by climate change – sediments may act as a source of phosphorus. Elevated water temperatures, coupled with reduced flow and water volume, can promote the development of anaerobic conditions in the sediment. These conditions enhance phosphorous release by lowering redox potential and promoting the dissolution of iron-bound phosphorus [46,47]. In contrast, during high-flow conditions, elevated oxygen levels and higher redox potential tend to limit phosphorus release, while particulate-bound material is more likely to be transported downstream. Additionally, elevated temperatures and low oxygen concentrations during low-flow conditions can stimulate denitrification, thereby reducing nitrate availability in the sediment [48]. However, the directionality of this association remains unclear for these studied rivers. It is uncertain whether sediment is acting as a nutrient sink through deposition (external eutrophication), releasing nutrients to the water column (internal eutrophication), or maintaining an equilibrium between the two processes [49].

Additionally, this relationship is further modulated by hydromorphological characteristics of rivers, such as flow velocity and sediment composition [50]. To clarify the mechanisms governing phosphorus exchange between sediment and surface water, future research should employ laboratory or mesocosm studies to monitor TP fluxes and other sediment-related biogeochemical processes. Our findings suggest that reducing surface water nutrient inputs alone may not be sufficient to improve macrophyte community composition – as required by the Water Framework Directive – because internally stored sediment nutrients can sustain eutrophic conditions and delay ecological recovery.

### Future directions for river restoration

In summary, our findings show a relation between sediment pore water TP in lowland rivers correlates with surface water TP concentrations. However, the underlying mechanisms – whether sediment is a source or sink for phosphorus – remain unresolved. Most species in the investigated lowland rivers are eutraphenic species, which explains the broad TP niches observed in our study. While, grey literature has reported similiar TP niches [51], to our knowledge published literature has largely overlooked this aspect.

If sediment nutrient saturation leads to broad macrophyte niches, species composition alone provides limited insight into the precise nutrient status of lowland rivers. Future studies could investigate potential ecological bottlenecks by conducting germination experiments to determine whether sensitive species can still re-establish and transplantation experiments to assess their competitive ability under current environmental conditions. Additionally, in channelized lowland rivers, restoration success may depend not only on nutrient reduction, but also on hydromorphological alterations. Effective restoration should integrate multiple approaches, including nutrient management, channel form adjustments, ecotone habitat restoration, and sediment stabilization. Given the high nutrient concentrations and constrained habitat availability in these systems, a combined strategy may be necessary to foster long-term improvements in macrophyte community structure and ecosystem health.

## Supporting information

S1 File Rationale for priors, (DOCX)
