# Supplementary material for: Macrophytes and their sedimentary phosphorus niche in lowland rivers
Source: PLoS One. 2025 Sep 2;20(9):e0330460. doi: 10.1371/journal.pone.0330460 (PMC12404402; doi:10.1371/journal.pone.0330460)
Supplement: S1 File — (DOCX) [file pone.0330460.s001.docx]

Supplementary material

**Macrophytes and their sedimentary phosphorus niche in lowland rivers**

**Prior formulation for pore water nutrients**

In 1988 Bloemendal and Roelofs published the book ‘Waterplants and Water Quality’ (English translation; 1988). This book was a compression of the report from Lyon and Roelofs (1986) available from: <http://edepot.wur.nl/386816>. This report contained tables on species and water quality in the pore water. In classes, from this table we searched the specific ‘weighted average’ (termed gg or in Dutch gewogengemmideld in the book). Since we need both information about the centrality (mean) and spread (standard deviation) for TP, the information on the desired species was extracted. For TP we used PO_4_^2-^ μmol L^-1^ in the pore water as a proxy, presented in table S1 below. For the ‘Total Mean’ and *Callitriche* spp. we calculated the weighted average and weighted standard deviation. Hence, if a species is not in the list (e.g., *Berula erecta*) we used the total mean as prior.

*Table S1: the values µ and SE used as priors.*

| Taxon | μ (original) | μ (75%) | SE | $\alpha_{\mu}$ | $\beta_{\mu}$ | $\alpha_{\sigma}$ | $\beta_{\sigma}$ |
| --- | --- | --- | --- | --- | --- | --- | --- |
| Berula erecta | 2066.25 | 1549.6875 | 239.8867 | 41.73263 | 0.02693 | 1 | 0.000645 |
| Callitriche spp. | 1717.125 | 1287.8438 | 128.4077 | 100.5876 | 0.078105 | 1 | 0.000776 |
| Ceratophyllum demersum | 3498.375 | 2623.7812 | 173.0565 | 229.8685 | 0.08761 | 1 | 0.000381 |
| Elodea canadensis | 1346.625 | 1009.9687 | 199.1522 | 25.7185 | 0.025465 | 1 | 0.00099 |
| Elodea nuttallii | 2372.625 | 1779.4687 | 121.1396 | 215.7788 | 0.12126 | 1 | 0.000562 |
| Myriophyllum spicatum | 3199.125 | 2399.3438 | 242.2791 | 98.07381 | 0.040875 | 1 | 0.000417 |
| Nasturtium microphyllum | 1282.5 | 961.875 | 457.8645 | 4.413299 | 0.004588 | 1 | 0.00104 |
| Nuphar lutea | 1197 | 897.75 | 217.5731 | 17.02553 | 0.018965 | 1 | 0.001114 |
| Potamogeton berchtoldii | 1638.75 | 1229.0625 | 428.293 | 8.235046 | 0.0067 | 1 | 0.000814 |
| Potamogeton crispus | 2543.625 | 1907.7188 | 237.5742 | 64.48076 | 0.0338 | 1 | 0.000524 |
| Potamogeton natans | 1090.125 | 817.5938 | 138.0513 | 35.0747 | 0.0429 | 1 | 0.001223 |
| Sparganium emersum | 1638.75 | 1229.0625 | 193.9785 | 40.14585 | 0.032664 | 1 | 0.000814 |
| Stuckenia pectinata | 4161 | 3120.75 | 184.7361 | 285.3741 | 0.091444 | 1 | 0.00032 |

Our study used TP μg L^-1^ and not PO_4_^2-^ μmol L^-1^ as in Lyon and Roelofs (1986). We multiplied the weighted average umol L^-1^ by 95 μg μmol^-1^ to obtain ug/L. TP is assumed to make up around ~75% of PO_4_^2-^ and was therefore multiplied by 0.75 to approximated TP in μg L^-1^. We multiplied it another time with 0.75 as we focus on riverine populations and riverine sediment has a lower TP concentration than lakes due to downstream transportation.

The standard deviation (SD) for each species was calculated as the SD over all the weighted averages (SDwa) in Lyon and Roelofs (1986) that occurred also in this study. The standard error (SE) for the individual species was calculated by SE=SDwa/sqrt(n) (n from Lyon and Roelofs (1986)). The priors for the mean were based on these calculated values as given in Tab S2 and explained below.

The assumption is that the data generating process (reflected by the samples) follows the behaviour of a gamma distribution. Therefore, the priors need to be specified with the alpha (α) and beta (β) parameters for the gamma distribution. The method of moments α=μ^2^/SE^2^ and β=μ/SE^2^ was used, obtaining these from Tab. S2. For the α and β parameters of SD α= μ^2^/μ^2^and β =μ/μ^2^ were used. The likelihood was also modelled as gamma distributed.

Likelihood:

$${for}_{sample}=\{i, \ldots, n\}$$

$$y_{sample}\sim Gamma(\alpha_{sample,taxa}, \beta_{sample,taxa})$$

$$\alpha_{sample,taxa}=\frac{{\mu^{2}}_{taxa}}{{\sigma^{2}}_{taxa}}$$

$$\beta_{sample,taxa}=\frac{\mu_{taxa}}{{\sigma^{2}}_{taxa}}$$

Prior parameters:

$${for}_{taxa}=\{i, \ldots, n\}$$

$$\mu_{taxa}=Gamma(\alpha_{\mu},\beta_{\mu})$$

$$\sigma_{taxa}=Gamma(\alpha_{\sigma},\beta_{\sigma})$$

**Prior formulation GLMM**

The priors were based on extracting information and fitting a GLM with Gamma distributed error and log link. This was performed using an intercept of -1.62 (SE=1.22) and a regression coefficient of 0.67 (SE=0.18) based on on Table 1 and 3 of Babu et al. (2000); additionally based on Fig. 2 from Talib et al. (2016) using an intercept of 0.31 (SE=1.78) and regression coefficient of 0.72 (SE=0.24). This resulted in the prior for the intercept N(-1, 1) and a regression coefficient of N(0.69, 0.14). Since there were multiple pore water samples at each river reach, which are not ‘truly’ independent, the variance was modelled at each site as a random effect. Since we log transformed the gradient used in the model we used the mean-log and both the studies and a wide standard deviation normally distributed as N(7.2, 7.2).

Likelihood:

$${for}_{sample}=\{i, \ldots, n\}$$

$$y_{sample}\sim Gamma(\alpha, \frac{\alpha}{\mu})$$

$$\log\left( \mu\right)=\beta_{0}+\beta_{1}*x_{sample,random}$$

Prior random effect:

$${for}_{random}=\{i, \ldots, n\}$$

$$x_{random}\sim Normal(7.2, 7.2)$$

Prior parameters:

$$\beta_{0}\sim Normal(-1, 1)$$

$$\beta_{1}\sim Normal(0.69, 0.14)$$

$$\alpha\sim Uniform(0, 2)$$
